# Unravelling the contributions of motor experience and conceptual knowledge in action perception: A training study

**DOI:** 10.1038/srep46761

**Published:** 2017-04-25

**Authors:** S. A. Gerson, M. Meyer, S. Hunnius, H. Bekkering

**Affiliations:** 1 Radboud University Nijmegen, Donders Institute for Brain, Cognition, and Behaviour, Nijmegen, The Netherlands; 2 Cardiff University, School of Psychology, Cardiff, United Kingdom; 3 University of Chicago, Department of Psychology, Chicago, USA

## Abstract

Prior knowledge affects how we perceive the world and the sensorimotor system actively guides our perception. An ongoing dispute regards the extent to which prior motor knowledge versus conceptual knowledge modulates the observation of others’ actions. Research indicates that motor experience increases motor activation during action perception. Other research, however, has shown that conceptual familiarity with actions also modulates motor activation, i.e., increased motor activation during observation of unfamiliar, compared to conceptually familiar, actions. To begin to disentangle motor from conceptual contributions to action perception, we uniquely combined motoric and conceptual interventions into one design. We experimentally manipulated participants’ experience with both motoric skills and conceptual knowledge, via motor training of kinematically challenging actions and contextual information about the action, respectively, in a week-long training session. Measurements of the effects on motor activity measured via electroencephalography (EEG) during pre- and post-training action observation were compared. We found distinct, non-interacting effects of both manipulations: Motor training increased motor activation, whereas additional conceptual knowledge decreased motor activation. The findings indicate that both factors influence action perception in a distinct and parallel manner. This research speaks to previously irreconcilable findings and provides novel insights about the distinct roles of motor and conceptual contributions to action perception.

Whether collaborating with, competing with, or learning from the social beings that surround us, we are constantly influenced by the actions of others and our interpretation of those actions. Given the importance of action perception, it is no wonder that the topic of how we perceive others’ actions, both in terms of behavioral correlates (e.g., action prediction and imitation) and neural correlates, has been a central debate across a variety of fields in recent years^1^,^2^,^3^. Research focusing on the neural correlates of action perception indicates that our own neural motor system is activated when we observe others’ actions^1^,^2^. The mechanisms underpinning the motor system’s response to the perception of others’ actions, however, are at the crux of an ongoing dispute.

Two factors have been found to modulate motor system activity during perception of others’ actions: *motor familiarity* and *conceptual familiarity* with the observed action. Our motor experiences are thought to facilitate predictions based on the precise kinematics of an action^4^, whereas our accumulated conceptual experience provides broader knowledge about the higher-order goals of an action, influencing predictions about an unfolding action sequence^5^. Yet, how motor and conceptual familiarity affect action perception and modulate sensorimotor activation in the brain is largely unknown. Here, we aim to disentangle motor from conceptual contributions to action perception.

## The Influence of Motor Experience on Action Perception and its Correlates

Intuitively, the link between motor experience and action perception appears simple: If we can perform a particular action, we are more likely to activate correlates of that action in our motor system when we see someone else perform it and, as a consequence, we can better understand the goal of the action. Support for this notion is now plentiful^1^,^2^,^4^,^6^,^7^. Correlational findings exist on both behavioural and neural levels for links between motoric experience and action perception. That is, both infants and adults who have more experience performing particular actions (relative to those who have less motoric experience) show a stronger response in their own motor system and better predict an action goal when perceiving the same action performed by someone else^2^,^4^,^6^,^7^,^8^,^9^,^10^,^11^. For example, Järveläinen and colleagues^9^ found that the degree of motor activity, as measured via decreased power in the beta rhythm, during the observation of goal-directed chopstick actions was related to the participants’ amount of experience using chopsticks. Building on these correlational findings, recent intervention experiments have manipulated participants’ motoric experience with motorically unfamiliar actions and found unique effects of motoric experience, relative to observational experience, on action perception in terms of both behavioural and neural measures^6^,^12^,^13^.

## The Influence of Conceptual Knowledge on Action Perception and its Correlates

Despite compelling evidence that the motor system is uniquely responsive to perceived actions with which individuals have motoric familiarity, contrasting findings indicating that the motor system is sometimes more responsive to actions with which individuals are *less* conceptually familiar question this general stance^14^,^15^. It has been proposed that conceptual information, information that is based on semantic or top-down knowledge, is one factor that underlies knowledge about how to use an object to achieve a certain goal^5^. Research examining the role of conceptual familiarity on neural correlates of action perception has found that the motor system (premotor, sensorimotor, and occipitotemporal regions commonly cited as part of the action observation network) is selectively activated during the perception of actions *contradicting* established conceptual knowledge, relative to conceptually familiar actions^14^,^15^. Cross and colleagues suggest that an increased response of the motor system during perception of conceptually unfamiliar actions may be a function of the motor system trying to create an action representation from little prior information^14^. In this way, it seems that the more difficulty participants had in predicting the ongoing sequence of actions (contradicting their conceptual knowledge), the more engaged the motor system was.

Developmental research has found relations between motor activation and conceptual knowledge during the perception of object-directed actions in the first year of life. In an electroencephalography (EEG) study, 12-month-old infants viewed actions that were predictable based on prior conceptual knowledge about objects and their typical action patterns (e.g., bringing a cup to the mouth) or were surprising in that they violated functional norms (e.g., bringing a cup to the ear^15^). In accordance with the findings by Cross and colleagues^14^, Stapel and colleagues found that the motor system was more active during observation of the actions that violated conceptual knowledge than during the more conceptually familiar, predictable actions. Thus, although motoric *familiarity* with an action increases activation of the motor system during action observation, *lack of familiarity* with an action in terms of its predictability on the basis of prior conceptual knowledge has the same effect on motor activation.

## Manipulation and Integration of Motor Experience and Conceptual Knowledge

At first glance, these different results appear to contradict one another. How is it that perception of familiar actions can lead to both an increase and decrease in motor activity? Due to the fact that researchers have largely investigated the effects of these different factors on action perception in isolation, little is known about how these independent findings can be reconciled. Are these findings truly in conflict or do changes in motoric and conceptual knowledge co-act to influence alterations in action perception independently via the motor system? The predictive coding framework addresses how these two factors might each uniquely influence action prediction^16^. In cases when greater motor activation is found during perception of motorically familiar actions, this perspective calls upon the fact that motoric experience allows particularly refined predictions about the unfolding scene, and these more refined predictions are thought to require more recruitment of the motor system^17^. With respect to greater motor activity during perception of unfamiliar actions that contradict conceptual knowledge, it has been hypothesized that familiar actions decrease the demands placed on the system for making accurate predictions principally due to decreased prediction errors^18^. Although this suggests that motor experience and conceptual knowledge might co-act and affect motor activity in parallel during action perception, this hypothesis has not yet been examined. Thus, in the current EEG training experiment, we directly examine this missing piece of the puzzle.

Although research examining individual differences in prior motoric experience or conceptual knowledge can convey information about relations between these factors, they do not permit causal interpretations. Therefore, we independently manipulated both factors, allowing us to examine their causal effects on the neural correlates of action perception within the same participants. In three training sessions, participants gained motoric experience with a motorically unfamiliar action (e.g., handling chopsticks). In addition, we manipulated the conceptual familiarity of an initially unfamiliar action outcome by altering prior predictions to fit with new contextual information. This manipulation made an otherwise atypical action outcome of a particular action more predictable and familiar (e.g. *this* person uses pliers to eat). In EEG measurements prior to and following the training sessions, motor activity during action perception was measured and indexed by power changes in the beta frequency band over central electrodes^9^. We took the difference in beta power between sessions as a measure of change in motor activity caused by the training sessions, such that a relative increase in power between sessions indicated a decrease in motor activity and a relative decrease in power indicated an increase in motor activity.

In both pre- and post-training EEG sessions, participants viewed actions in which a hand picked up a tool (fork, chopsticks, pliers, or wrench) and used it to act on one of two presented items (food or workbench). Each type of tool was a priori classified as motorically familiar (fork and pliers) or unfamiliar (chopsticks and wrench) and could be paired with a conceptually matched (e.g., fork/chopsticks to food) or mismatched (e.g., fork/chopsticks to workbench) goal-item (i.e., action outcome). During motoric training, participants practiced using either the chopsticks or the wrench to improve their performance on one of the two motorically unfamiliar tool actions (henceforth referred to as *trained*). The other of these two tools remained classified as motorically unfamiliar. To manipulate conceptual familiarity and, consequently, the predictability of the observed action outcome, we manipulated the context of a particular observed action (*manipulated*) and thereby the tool-goal-item association. Participants were asked to memorize information regarding a person who used one type of tool in an atypical manner (e.g., used pliers to eat) in a particular context (e.g. in an art project). The other atypical tool-item pairing (e.g., fork to workbench) was not manipulated and remained a conceptual violation as the observed action unfolded (unfamiliar). We assessed the difference in motor activity across sessions (indexed by beta power; see Methods) to the motorically familiar, trained, and unfamiliar actions and to the conceptually familiar, manipulated, and unfamiliar action-item pairs.

We hypothesized that motor activity would increase across sessions for the motorically trained action and decrease for the conceptually manipulated action sequence as the action unfolded in a conceptually unfamiliar manner. If motoric experience and conceptual knowledge interact, we would expect an interaction between these two variables such that motor activity would only increase for the motorically trained action when viewing the conceptually familiar form of this action (e.g., chopsticks acting on food rather than on workbench) and motor activity would only decrease for the conceptually manipulated item when viewing the motorically familiar action (e.g., pliers instead of wrench acting on food). If, however, these two types of experience act independently, we should find main effects of motoric and conceptual experience and no interaction between the two.

## Results

**Figure 1:**
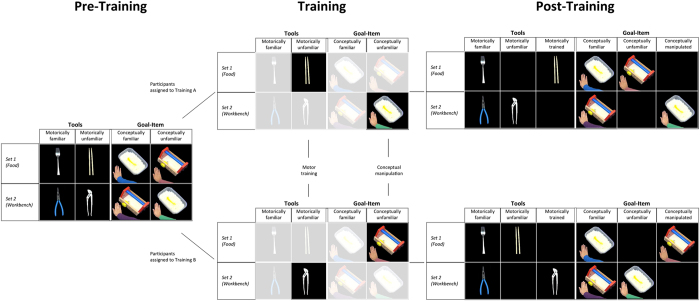
This schematic illustrates the stimuli and manipulations associated with the Pre-Training, Training and Post-Training phases. Pre-Training phase: Tools which were a priori motorically familiar (like fork and pliers) and unfamiliar (like chopsticks and wrench) were used for actions on goal-items that were, when paired, conceptually familiar (e.g. fork with food) or unfamiliar (fork with workbench). Each tool and goal-item combination was uniquely associated with a particular shirt color. This association was not made explicit to the participants. Training: Participants were randomly assigned to one of two Training groups. Training group A received motoric training with using the motorically unfamiliar tool from Set 1 (chopsticks) and were assigned to a conceptual manipulation that makde an initially conceptually unfamiliar tool-goal-item pairing conceptually familiar (Set 2- tools [e.g., pliers] with food). For Training group B, the sets for the motoric training and conceptual manipulation were reversed. Post-Training: For each Training group, two new categories emerged, the motorically trained tool and conceptually manipulated tool-goal-item association. In the analyses of interest, we collapsed over the stimuli across training groups to contrast motorically familiar, unfamiliar, and trained as well as conceptually familiar, unfamiliar, and manipulated actions.

To allow the opportunity to investigate the neural response to the action as it unfolded, we segmented each action video that participants viewed during the EEG sessions into time segments based on sub-actions (e.g. lifting a tool, adjusting grip on the tool, acting with the tool on the goal item [i.e., tool box or food]; see Fig. 1). We conducted an initial Generalized Estimating Equation (GEE) with beta power difference between the pre- and post-training sessions as the dependent variable and motor familiarity (motorically familiar, trained, or unfamiliar), conceptual status (conceptually familiar, manipulated, or unfamiliar), and time segment (1–5) as the within-subjects factors. There was no evidence that motoric familiarity and conceptual familiarity had interacting effects (*p* = 0.92), so separate analyses were conducted for each of these factors. For simplicity, we further refer to a decrease in beta power across sessions as increased motor activity and an increase in beta power across sessions as decreased motor activity.

**Figure 2:**
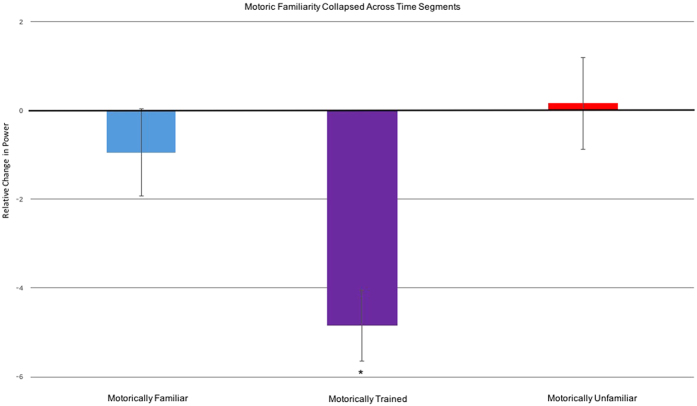
Across sessions, there was a decrease in power for the motorically trained action but not for the motorically familiar or unfamiliar actions (*p < 0.05).

An analysis of motor familiarity revealed main effects of motor familiarity, χ^2^(2) = 45.26, *p* < 0.001, and time segment, χ^2^(4) = 23.08, *p* < 0.001, but no interaction between motor familiarity and time segment (*p* = 0.89). The main effect of time segment was driven by an increase in motor activity in later, relative to earlier, time segments. Given that the later time segments involved movement, relative to a still frame, this is unsurprising. Consistent with our hypothesis, the effect of motor familiarity was driven by increased motor activity across sessions during perception of the motorically trained action, relative to the motorically familiar (*mean difference* = 3.90, *SEM* = 1.09, *p* < 0.001) or motorically unfamiliar action (*mean difference* = 5.01, *SEM* = 0.84, *p* < 0.001; see Fig. 2, collapsed across time segments).

**Figure 3:**
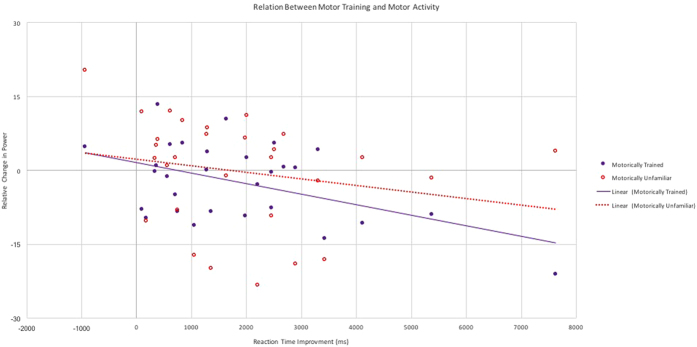
Improvement in behavioural efficiency (positive values on the y-axis represent faster reaction times) were related to relative changes in beta power (from pre- to post-training) when observing the trained action but not when observing the unfamiliar, untrained action.

We further explored individual differences in the effectiveness of motoric training (i.e., a behavioral index of motor learning) on variability in the altered motor activity during perception of the motorically unfamiliar and trained actions. We examined the mean difference in motor activity between pre- and post-training sessions for perception of each action initially defined as motorically unfamiliar (i.e., wrench and chopsticks) and assessed how this activity related to motoric improvement (measured behaviorally) across training sessions in learning one of these two actions (as defined by decreases in reaction time throughout training when performing the trained action). When both motor activity during perception of trained and unfamiliar actions were entered into a linear regression with reaction time (during action production) as a dependent variable, it revealed a significant effect for motor activity *only* during perception of the motorically trained action (*B* = −0.46, *p* = 0.01, ∆R^2^ = 0.21, *p* = 0.01), indicating that individual differences in behavioral improvement during training related to individual differences in changes in motor activity to this specific action when perceived during pre- versus post-training (see Fig. 3). For example, individuals who greatly improved in their ability to use chopsticks showed a particularly large increase in motor activity across sessions when viewing this action (but not the wrench action).

**Figure 4:**
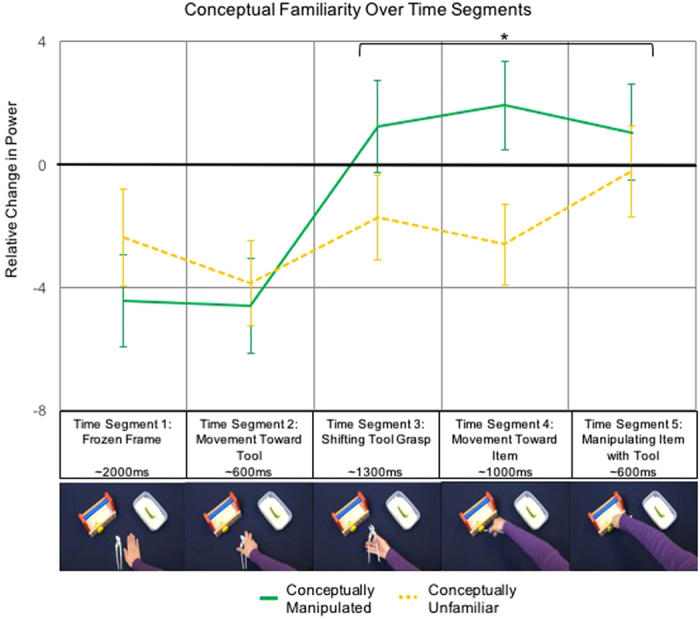
Across sessions, there was an increase in power for the conceptually manipulated action, relative to the conceptually unfamiliar action, for time segments 3–5 (*p < 0.05). The photographs beneath the time segments depict example still frames of the event that defined each period. The goal-item toward which the actor moved with the tool became clear between time segments 3 and 4. The time segments were matched in duration across all trials.

The analysis of conceptual familiarity revealed a marginal main effect of conceptual status, χ^2^(2) = 4.92, *p* = 0.086, a significant main effect of time segment, χ^2^(4) = 20.68, *p* < 0.001, and an interaction between conceptual status and time segment, χ^2^(8) = 15.89, *p* = 0.04. Exploring the effect of conceptual status across time segments (see Fig. 4) indicated that the differences in conceptual status largely occurred in the latter half of each trial. This is in line with the fact that the tool-goal-item association (i.e., action outcome) that was being manipulated was only apparent from time segment 3 or 4 (when the tool changed directions toward the goal-item). We followed up by examining the effect of conceptual status over time segments 3–5 (when it became apparent that the tool was approaching a specific goal-item and, thus, when updating would have originally occurred during online processing). This analysis revealed a significant effect of conceptual status, χ^2^(2) = 11.20, *p* = 0.004, a significant main effect of time segment, χ^2^(2) = 6.20, *p* = 0.045, and no significant interaction (*p* = 0.69). The main effect of conceptual status was a function of a relative decrease in motor activity across training sessions during perception of the manipulated tool-goal pair (i.e., the pair that previously violated conceptual knowledge but was manipulated to be familiar in the given context) relative to the unmanipulated, conceptually unfamiliar action that violated conceptual knowledge (*mean difference* = 2.96, *SEM* = 0.89, *p* = 0.001).

## Discussion

This experiment showed that an increase in motor familiarity led to a relative increase in motor activity across sessions during observation of the trained action. Interestingly, greater improvement in performing this action directly related to greater changes in motor activity measured across sessions. Providing conceptual information to make a participant familiar with a previously conceptually unfamiliar action, on the other hand, resulted in a relative decrease in motor activity during perception of the action that initially violated conceptual knowledge. Importantly, these two effects were found distinctively in parallel without a significant interaction between these two effects.

Together, these findings suggest that motor familiarity with the kinematics of an observed action and conceptual familiarity with an action outcome simultaneously, and in an independent manner, modulate motor activation during action perception. We propose that these factors work in parallel such that motor experience likely leads to the generation of more precise predictions within the sensorimotor system, whereas conceptual knowledge influences the updating of predictions in the sensorimotor system as an observed action is unfolding.

According to the predictive coding framework, predictions about observed actions are continuously generated and updated based on prediction errors in order to accommodate one’s internal model of the world^16^. A predictive model can generate predictions based on a variety of different sources. In the case of motor learning, predictions (often referred to as proprioceptive predictions^17^) about a particular motor act become more detailed with increased motoric expertise. This is hypothesized to be reflected in enhanced motor activity, as reported here for the trained action following training [see also refs 7,12,18]. During the observation of others’ actions, predictions are generated based on associations between current sensory input and upcoming motor programs^16^. That is, an observed action (e.g. lifting chopsticks) is linked with a subsequent motor plan (e.g. contracting the finger muscles to bring the front ends of the chopsticks towards one another in a pincer grasp). The more extensive one’s own motor experience is, the more detailed the generation of the motor planning becomes and, consequently, the more precise the respective predictions that can be generated. This increase in precision is reflected by an increase in motor activation during observation of actions within one’s own motor repertoire. Our findings are consistent with this perspective and show that relative improvement in motoric abilities is directly related *and* specific to the particular action trained.

Once generated, each prediction can be compared to the subsequent sensory input and updated with regards to that input in terms of exteroceptive predictions^19^. For example, if a prediction does not match the subsequently observed movement, a prediction error is signalled that allows for a prediction update^20^. Previous work suggests that the need to update predictions in an action observation context is reflected in enhanced activity of the motor system^16^,^17^,^18^,^19^,^20^,^21^. Providing additional information about the action context (i.e., manipulating conceptual information) in the manipulated condition of the current study allowed the participants to update the existing model of the action sequence prior to action observation, such that an initially surprising action that violated conceptual knowledge (during the pre-training session) subsequently became familiar and predictable (during the post-training session) through incorporation into conceptual knowledge about the action within a particular context. Due to the updated model, the motor system likely generated more accurate predictions during action observation in the post-training session, thus reducing the need for updating, as reflected in the relatively reduced motor activity following the manipulation of conceptual information. This interpretation is consistent with the interaction between conceptual familiarity and time segment we saw in the motor activity decrease between sessions. During the pre-training session (and for the unfamiliar actions that were not manipulated, during the post-training session), an updating of the model was likely needed once the tool began to approach the goal-item that violated conceptual knowledge (e.g., when the pliers approached the food; first perceivable at the beginning of time segment three). Following the conceptual manipulation, however, such updating was not necessary for the manipulated action during viewing because the updating had occurred prior to viewing. Thus, the change seen for this action (decreased motor activity across sessions) was during the period (time segments three to five) when updating would have originally occurred during online processing.

The current findings are consistent with and extend prior research from two perspectives that were previously considered irreconcilable^10^,^11^,^14^,^15^. They suggest that the motor system is simultaneously and differentially sensitive to changes in both motor and conceptual familiarity. This research focused on motor activity as reflected by power suppression in the beta rhythm, based on previous research on action execution and observation^9^,^22^,^23^. Suppression in the beta frequency band during action processing (execution and observation) is associated with origins in premotor and primary motor areas^24^. It should be noted that besides beta suppression, suppression in alpha power over sensorimotor regions (also called mu rhythm) has also been associated with motor activity^2^,^11^,^12^. Compared to beta band activity, mu suppression is associated with more posterior brain regions^24^ and is often reported in developmental research^11^,^12^,^15^. Recent findings suggest developmental and functional differences between mu and beta suppression^25^,^26^. Future research should systematically investigate the differential functions of alpha and beta frequencies over motor regions in action perception.

The manipulation of experience for adults in this research raises questions concerning natural changes in experience that occur throughout development. For example, previous research has highlighted the role of motor experience on increased motor activity in infants^6^,^11^. In contrast, other research has demonstrated that infants’ motor systems are more active during the perception of actions that violate conceptual knowledge, relative to perception of conceptually familiar actions^6^. The present research suggests that these changes seen in typical development may be a function of two independent mechanisms and that changes in motor experience versus conceptual knowledge should lead to distinct modulations in the motor system throughout the lifespan, but replications and extensions of the current findings with developmental samples and methodologies that allow source localization (e.g., magnetoencephalography) will help clarify the functional mechanisms underlying these adaptations in action processing.

## Materials and Methods

### Participants

Thirty-two participants (21 female, age range: 18–40 years) took part in this EEG study. Seventeen participants were assigned to receive motor training with chopsticks (Training Group A; see Fig. 1) and fifteen were assigned to receive motor training with a wrench (Training Group B). Participants were recruited in Nijmegen, a middle-sized urban city in the Netherlands. The majority of participants were students at the university, and they received either a gift voucher or study points for their participation. All experiments were carried out according to standard guides and regulations and were approved by the ethics committee at Radboud University Nijmegen. Participants all completed written informed consent prior to participation.

### Stimuli and Equipment

#### Videos

We created a set of 128 unique videos. In each video, a hand first rested next to a tool between two goal-items (a workbench with a green or yellow screw and a bowl of rice with a green or yellow pepper; see Fig. 1). After a 2000 ms still frame (Time Segment 1), the hand reached for (Time Segment 2), picked up (Time Segment 3), and moved the tool toward one of the two goal-items (Time Segment 4). If the hand with the tool reached for the workbench, the tool was used to turn the screw approximately 180 degrees (Time Segment 5). If the hand with the tool reached for the food, the tool picked up the pepper and some rice (Time Segment 5). See Fig. 4 for a depiction of these time segments. Eight different tool-goal-item pairs were possible, with each of the four tools (fork, chopsticks, plier, and wrench) reaching toward each of the two goal-items an equal number of times. Across these eight combinations, each combination of green and yellow screws and peppers were filmed and the goal location was counterbalanced between the left and right side. For each of these 64 combinations, we filmed two versions to introduce minor natural variability in the stimuli, resulting in 128 unique videos. The timing of each video was closely matched using a metronome. Four different colored shirts were worn in the videos, as described below in the *Conceptual Manipulation Stimuli* section (see also Fig. 1).

#### Catch Trials

In order to maintain participants’ attention to both the tool and the object on which it was used to act, 10% of trials were followed by catch trial questions, distributed pseudorandomly across all trials. On catch trials, an image appeared following a video. In the image, participants saw a tool (e.g., a wrench) and a goal-item (e.g., a yellow pepper food object), and they were asked to respond as to whether they saw the tool act on the pictured goal-item in the last video via a button press.

#### Conceptual Manipulation Stimuli

In order to manipulate participants’ conceptual familiarity with the pairing of tools and goal-items, participants read stories (i.e., conceptual prompts) about four individuals during each training session. Three of the stories were control stories, in which a person was identified by shirt color and some information was given about her, including her name, favorite color, studies, and hobbies. The fourth story gave specific information identifying a reason why that individual (identified by shirt color) used either a fork to act on a workbench (i.e., The girl in green is working on a lifestyle art project in which she uses cutlery instead of tools to build things; given to participants motorically trained with a wrench; Training Group B) or pliers to eat (i.e., The girl in purple is working on a lifestyle art project in which she uses tools instead of cutlery to eat her food; given to participants motorically trained with chopsticks; Training Group A).

#### Motor Familiarity Measures

To ensure that our assumptions concerning participants’ relative motoric familiarity with the fork, chopsticks, pliers, and wrench were justified, we asked all participants to complete a questionnaire asking them about their experience with each type of tool. As expected, participants said they had used forks and pliers more recently and more frequently than chopsticks and wrenches, respectively. On a 1–5 scale, frequency ranged from 1 (never) to 3 (once a month) for both chopsticks and wrench use (note: we screened to rule out frequent chopstick use during recruitment).

**Figure 5:**
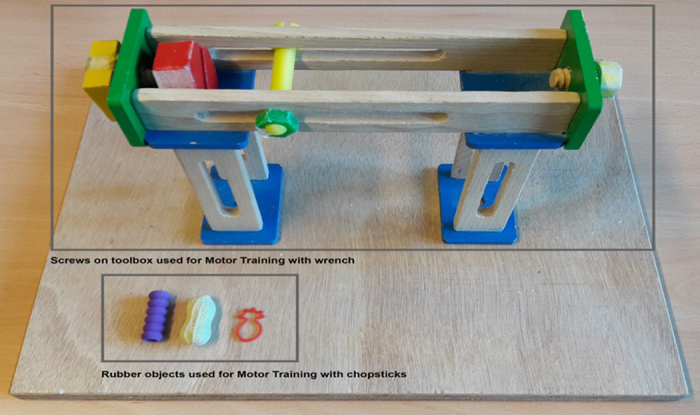
During motor training, those participants trained with the wrench turned each of three screws on the above object for 20 trials each from easiest (square yellow screw, far left) to hardest (octagonal green screw, center front). Participants trained with the chopsticks practiced moving each of the rubber objects depicted above to a different location for 20 trials each from easiest (peanut, middle) to hardest (pineapple, right).

At the beginning of each EEG session, all participants performed one action with each tool. The four tools were identical to those seen in the video. The fork and chopsticks were used to pick up a small rubber objects (see Fig. 5) and move them to a different location. The pliers and wrench were used to turn a screw on a workbench. As expected, participants took longer to perform the chopsticks action than the fork action in the first session (mean reaction times approximately 2040 ms and 2800 ms, respectively; *p* = 0.007) and descriptively longer to perform the wrench action than the pliers action (mean reaction times approximately 3440 ms and 5145 ms, respectively; *p* = 0.067). During training sessions, participants either practiced with the chopsticks or the wrench. They had the chance to perform the designated action up to 60 times per session (or as many times as possible within 15 minutes), using 6 different items (rubber items or screws) that increased in difficulty throughout the session (see Fig. 5).

### Procedure

Each participant attended five sessions spanning a week’s time. The first and last sessions both involved action production and action observation during EEG recordings and lasted approximately two hours each. The three intervening sessions were half hour training sessions.

#### EEG Sessions

At the beginning of the session, participants completed informed consent and were fitted with a lycra EEG cap with 28-channels. Following cap placement, participants were given instructions for completing the action task. They pressed a button prior to picking up each tool and after returning to the tool to its original position so that reaction times could be measured. They always completed the actions with the fork and chopsticks, followed by the actions with the pliers and wrench.

Following the action session, they were told the instructions for the video session (i.e., how to answer catch trials). Sitting about 75 cm distance from the screen, they then watched 8 blocks of video clips, consisting of 422 videos in total. Participants were allowed breaks in between each block and could press a button when they were ready to continue. Videos were presented in a pseudorandomzied order such that there were at least 8 trials between repetitions of the same video, the same tool (chopstick, fork, wrench, pliers) could occur maximally twice in a row, and the same goal-item (food, workbench) could occur maximally four times in a row. The only difference between the first and second EEG session was that, in the second session, participants were asked to recall details about the conceptual prompts prior to the video session (following action production) and to reread the conceptual prompts between blocks.

#### Training Sessions

**Figure 6:**
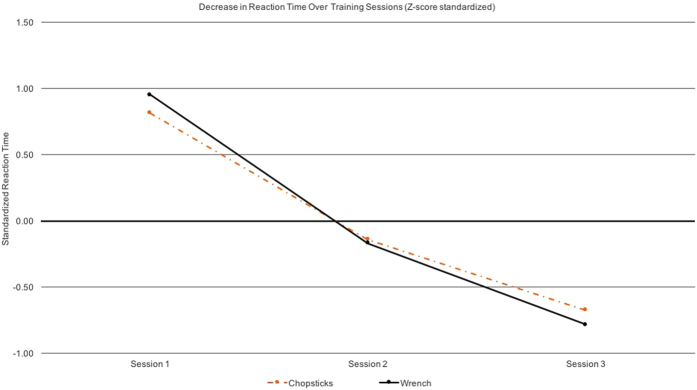
Both participants trained to use the wrench and chopsticks improved in their reaction times across training sessions. Due to the fact that, across training sessions, reaction times differed between the chopsticks and wrench actions, the change in reaction times is plotted above in terms of Z-transformed scores.

During training sessions, each participant practiced with either the chopsticks or the wrench, based on random assignment. They were asked to practice the action 20 times each with each of three different objects (either rubber objects or screws depending on the assignment) for a maximum duration of 15 minutes. The participants were asked to position their hand on the start location (i.e. response button) and to fixate on a fixation cross presented on the screen until it disappeared. Then participants had to lift their hand from the start location and execute the action. For the participants who trained with the wrench, this entailed picking up and lifting the wrench, adjusting their grip such that the wrench fit the respective screw size, using the wrench on the screw and turning the screw 180 degrees, setting down the wrench and bringing the hand back to the starting button (the press of which acted as a response time measure). For participants who trained with the chopsticks, this entailed picking up and lifting the chopsticks, using the chopsticks to pick up the rubber object which was positioned in a box of dry rice, and transporting the rubber object to a second compartment of the box. To finish, participants had to set down the chopsticks in their starting location and bring their hand back to the starting button. All participants were asked to execute the actions with their right hand only (prerequisite for participation in the study was being right-handed) and as quickly and accurately as possible. All participants, regardless of condition, improved in reaction times throughout the three training sessions (see Fig. 6). Following motor training, participants read each of the four stories about the actors at least once (but were told they could read them as again if needed). They were told to take their time reading each of the stories.

### Data Processing

#### EEG recordings

We recorded from twenty-eight active electrodes arranged in the 10–20 system with an online reference to the left mastoid. Additionally, eye-movements were recorded with four electrodes arranged vertically, above and below the left eye as well as horizontally next to the eyes. The signal was amplified using a BrainAmp DC EEG amplifier, band-pass filtered (0.1–125 Hz), and digitized at 500 Hz. Impedances were kept below 20 kΩ.

#### EEG analysis

**Figure 7:**
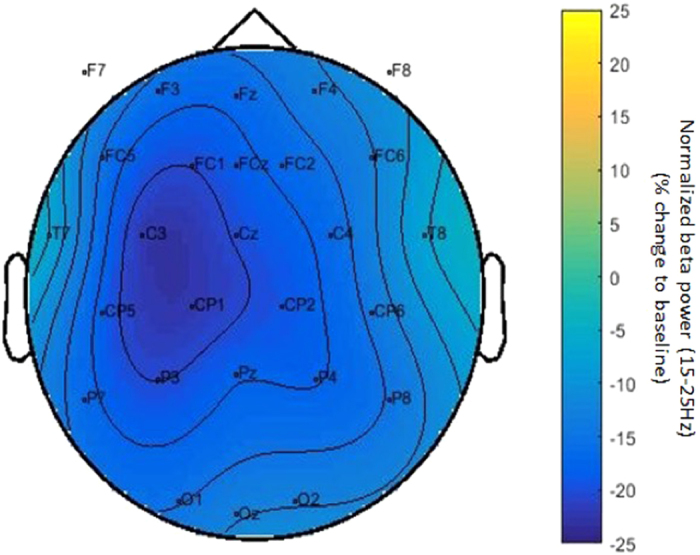
Topography of normalized beta-power, averaged across all action observation conditions of both EEG sessions.

For data analysis we used FieldTrip, an open source Matlab (version 7.0, TheMathWorks, Inc.) toolbox developed at the Donders Institute for Brain, Cognition and Behaviour^27^. For each session, the data were initially segmented into trials that were time-locked to the onset of movement in the videos. The trial began two seconds before the movement began, when a still frame was presented, and ended four seconds after the movement started (after the tool had made contact with and begun to manipulate the goal-item). In preprocessing, line noise was removed, and the data was rereferenced offline to the linked mastoids. Segments were inspected visually to exclude excessive EEG artifacts before applying ICA to remove eye-movement and heartbeat components. Subsequently, the data were once again inspected visually to exclude any remaining artifacts. We used a Fourier analysis with a 1 second sliding Hanning taper and spectral smoothing of 3 Hz to calculate the time-resolved frequency representation of power between 1 and 50 Hz. The power estimates were then baseline-corrected to the fixation cross period preceding the beginning of the trial (−3 to −2 sec relative to movement onset) by calculating the relative percent of change from baseline to each data point. We then pooled the data across both sessions and all conditions to investigate the sample-specific topography and to identify the electrodes of interest for further analysis. The topographic results for the beta frequency range (15–25 Hz) are presented in Fig. 7. As expected from previous research^22^, beta power decreased with respect to baseline during the observation of others’ actions. Beta suppression during action observation was strongest over electrodes C3 and CP1 in this sample (see Fig. 7). These electrodes were thus used in all subsequent analyses. The anticipatory beta suppression during the still frame (−2 to 0 sec) and further drop in beta power from the onset of movement observation (at 0 sec) is consistent with previous studies indicating motor activity during prediction and observation of actions^9^,^22^,^23^. For the analysis of changes in power between the two sessions, we then split the data by session and condition and calculated the difference in power between sessions per participant (Session 2 – Session 1). Data were then pooled over 15 to 25 Hz (beta frequency range) and the electrodes C3 and CP1 as determined by the topographic distribution. To additionally account for the unfolding of the sub-actions in the stimulus (i.e. still frame: −2 to 0 sec; movement towards tool: 0 to 0.6 sec; shifting tool grasp: 0.6 to 1.9 sec; movement towards goal item: 1.9 to 2.9 sec; manipulating goal item with tool: 2.9 to 3.5), we separated the difference values into five separate time segments. Difference values were then entered in the statistical comparisons between conditions and time segments using Generalized Estimating Equations.

## Additional Information

**How to cite this article:** Gerson, S. A. *et al*. Unravelling the contributions of motor experience and conceptual knowledge in action perception: A training study. *Sci. Rep.* **7**, 46761; doi: 10.1038/srep46761 (2017).

**Publisher's note:** Springer Nature remains neutral with regard to jurisdictional claims in published maps and institutional affiliations.
